# Mixed Micellar Solubilization of Naphthol Green B Followed by Its Removal from Synthetic Effluent by Micellar-Enhanced Ultrafiltration under Optimized Conditions

**DOI:** 10.3390/molecules27196436

**Published:** 2022-09-29

**Authors:** Amnah Yusaf, Muhammad Usman, Muhammad Siddiq, Manahil Bakhtiar, Asim Mansha, Saadia Shaukat, Hafiza Fatima Rehman

**Affiliations:** 1 Department of Chemistry, Government College University, Faisalabad 38000, Pakistan; 2 Department of Chemistry, University College London, London WC1E 6BT, UK; 3 Department of Chemistry, Government College Women University, Faisalabad 38000, Pakistan; 4 Department of Chemistry, Quaid-i-Azam University, Islamabad 45320, Pakistan; 5 Department of Chemistry, Hazara University, Mansehra 21120, Pakistan; 6 Department of Chemistry, Government College Women University, Sialkot 51310, Pakistan; 7 Department of Zoology, Government College University, Faisalabad 38000, Pakistan

**Keywords:** NGB, surfactants, partition coefficient, Gibbs free energy, rejection percentage, permeate flux

## Abstract

In this manuscript, the application of cetyltrimethylammonium bromide (CTAB) and cetylpyridinium chloride (CPC) for the removal of Naphthol Green B (NGB) as a synthetic effluent has been studied. The solubilization of NGB by a single and mixed micellar system using Triton X-100 (TX-100) as a nonionic surfactant has been performed to establish both the extent of the partitioning (k_x_) of NGB and ultimately their respective Gibbs free energies ΔG_p_ as well. An applied methodology, micellar-enhanced ultrafiltration (MEUF), has also been studied in different micellar media of cationic surfactants by variation in some selective parameters, such as the concentration of surfactant, electrolyte, pressure, pH, and RPM to obtain optimum conditions. The results have been analyzed by a UV/visible double beam spectrophotometer. ΔG_p_ was found to be −39.65 kJ/mol and −47.94 kJ/mol by CTAB and CPC, respectively, in the presence of a nonionic surfactant. The maximum value of Gibbs free energy (ΔG_p_) of the partition was obtained by CPC. The values of the rejection coefficient (R%) and permeate flux (J) are also calculated. A maximum removal of 99.77% and 98.53% by CTAB and CPC, respectively, was obtained. It has been observed that both of the surfactants are strong candidates for NGB removal.

## 1. Introduction

The textile industry is considered one of the major contributors to anthropogenic activities and is responsible for building the global economy, contributing to environmental pollution. Currently, 7 × 10^7^ tons of dyes are synthesized each year, from which textile industries utilize about 10,000 tons, while the rest is used by paper, food, pharmaceuticals, cosmetics, and other industries. Therefore, it is the need of the hour to develop some novel materials for the treatment of colored wastewater [1].

Poor disposal of dyestuff into the water bodies by these industries is a major concern these days. Excessive use of dyes in textiles at various stages (such as softening, brightening, sizing, desizing, and finishing agents) produces highly colored wastewater in which the presence of carcinogenic and mutagenic chemical entities causes serious health hazards [2]. Therefore, an efficient and long-term sustainable wastewater treatment technique should be established to resolve this issue. Pollutants such as dyes should be properly filtered before being discharged into the environment and should be taken as a serious hazard to human health and the environment [3]. Various physical (adsorption) [4], chemical [5], and biological photo degradation (enzyme degradation) [6] methods are applied for effluent treatment with varying efficiency and removal percentage rates.

Many of these methods being used for dye removal are not applicable for various reasons. The oxidation technique is less efficient and needs significant dilution, degradation and adsorption are expensive and time-consuming, reverse osmosis requires high-pressure values and still lower permeate flux, while others are less efficient, with high conducting costs or producing large amounts of sludge [7]. The development of membrane technologies has been found to be an efficient and affordable solution for water treatment due to low cost, high efficiency, and easy operation in food, pharmaceuticals and especially textile industries [8,9].

A variety of membranes were developed using biomaterials, and polymers have potential applications for water treatment [10]. Comparatively, one of the most efficient and feasible methods is a membrane-based separation technique, such as micellar-enhanced ultrafiltration (MEUF) This technique combines the high flux of ultrafiltration (UF) and the high selectivity of reverse osmosis (RO). MEUF involves the use of surfactants owing to some of their outstanding properties. Surfactants, due to their amphiphilic nature, can solubilize compounds which otherwise would not be soluble and are also used as a wetting agent. They congregate to form micelles after acquiring critical micelle concentration (CMC) [11].

An advantage of micellar-enhanced ultrafiltration over other techniques is that it is considerably a better method than the usually available membrane-based separation processes due to easy operating system, low energy consumption and high removal productivity. The nature of the membrane used in MEUF can also affect the removal efficiency.

A disadvantage is the high membrane cost and slow speed filtration, also membrane fouling may occur as the pores of the membrane become blocked due to prolonged uses, but the membrane can be reused after regeneration and requires a regular cleaning process. At large scale, membranes with relatively large pore size may be used in order to obtain larger flux [12].

The fundamental theory of MEUF was initiated by McBain and Jenkins in 1922 for anionic surfactants and by H. Schott in 1964 for nonionic surfactants. However, Scamehorn publicly proposed MEUF in several research publications in the 1980s. This methodology is the best one available in the present day since it can trap particles at even extremely low concentrations by micellization caused by ionic or hydrophobic contact between them [13]. In this process, many proteins, polynuclear hydrocarbons, and dyes are stabilized by these micelles’ formation. Generally, micellar aggregates range in size from 1–10 nm which makes ultrafiltration membranes unable to remove them easily. The dye and surfactant interaction enables the ultrafiltration membrane in MEUF to remove these dye molecules readily from an effluent by entrapping the dye molecules into the micelles and resultantly increasing their size. The pollutants are either affixed at the micellar surface (metal ions) or to its inner core (dye stuff, etc.). Solute-entrapped micelles are rejected from fluid downstream (permeate), while in feed (retentate), the concentration of solute and surfactant increases. Micelles containing solubilized solutes are filtered by the UF membrane by size exclusion mechanism [14].

In reported work, cationic surfactants, i.e., cetyl trimethyl ammonium chloride (CTAB) and cetyl pyridinium chloride (CPC), are used against negatively charged dye. CTAB has been extensively used in the production of inorganic materials as a structure-directing agent, passivating agent, coating agent, and stabilizing agent and also assists in the deposition of target components. It is frequently employed in wastewater treatment due to its better absorption capacities. Adding CTAB to materials improves their sensitivity and limit detection [15]. CPC is a quaternary ammonium cationic surfactant with broad-spectrum antiseptic properties. It is used in cosmetics and pharmaceutics as an antimicrobial preservative. It is utilized therapeutically as the antiseptic agent to treat catarrhal inflammation of mucous, oral, nasal, and throat infections by combining it with other medications or by use on its own [16].

In the present study, an experimental investigation has been undertaken to filter the dye Naphthol Green B (NGB) from an aqueous stream by using an ultrafiltration cell based upon MEUF). The aqueous solution of NGB has an intensive dark green color and hence causes adverse effects on aquatic organisms and the environment. The retention characteristics and permeate flux were studied in detail under the effect of different operating factors. The performance of the process was determined by dye rejection and flux profile. In MEUF, surfactant concentration must be well above its CMC.

The current study aimed to elaborate on the potential of nonionic surfactant TX-100 and cationic surfactants CTAB/CPC in synergism for the removal of anionic dye Naphthol Green B. This work has novelty because the industrial effluents already contain surfactants, as do some washing units inside using soaps, so if an industrialist can only install a membrane setup at the outlet, results would be exceptionally impressive. The expectations from this study include a better understanding of the mechanism and potential of micellar-enhanced ultrafiltration for dye removal which will further enhance the chances of resolving challenges in dispersion technologies.

## 2. Results and Discussion

### 2.1. Absorption Spectroscopic Study

#### 2.1.1. Interaction of NGB with CTAB and CPC

**Figure 1:**
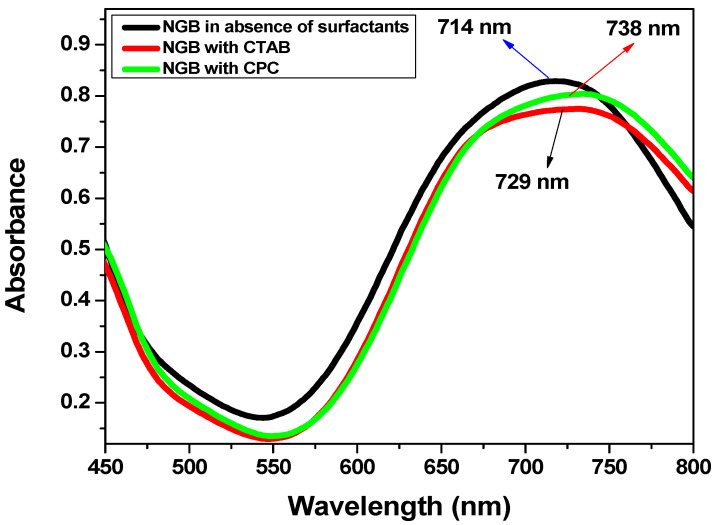
Spectra of simple absorption of NGB with and without surfactant.

Figure 1 displays the absorption spectra of Naphthol Green B before and after surfactant addition. The solution with only Naphthol Green B has a maximum absorbance of 714 nm, showing no difference from the reported value for this dye [17]. Surfactant addition shifted the absorption peak from 714 nm to 738 for CPC and 729 for CTAB at a fixed concentration of NGB, as in Figure 1. This shift is called red or bathochromic shift and is a strong indication of NGB–surfactant interaction [18] caused due to the electrostatic and hydrophobic interactions between the molecules of NGB and surfactants (CTAB and CPC). Such interactions normally occur when the concentration of surfactant is lower than the critical micellar concentration and is called the limiting association concentration (LAC) [19]. A bathochromic (redshift) in the spectra of NGB with surfactant depicts the strong host–guest association between anionic dye and cationic surfactant. This bathochromic shift in the spectrum may be the result of solvatochromism. This is a process in which the absorption spectra of additives shift towards a higher wavelength due to a decrease in solvent polarity and ultimately lead to the redistribution and restabilization of energy levels [20].

**Scheme 1:**
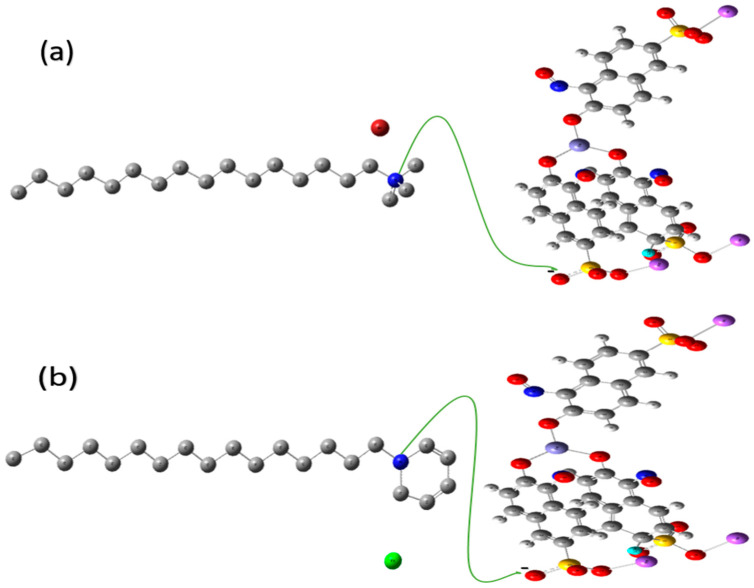
Naphthol Green B binding with (**a**) Cetyltrimethyl ammonium bromide, (**b**) Cetyl pyridinium chloride.

As NGB has an anionic polar group, strong interactions take place between the cationic surfactant and anionic dye. This bathochromic shift is observed from 714 to 729 nm due to the electrostatic and hydrophobic interactions of alkyl chains of CTAB and aromatic rings of cationic dye. The hyperchromic effect was noticed in the post-micellar region due to an increase in surfactant concentration, indicating strong interactions. This hyperchromic effect is due to an increase in the absorption intensity caused by a substituent [20]. Similarly, in the case of CPC, the λ_max_ was observed toward a longer wavelength of 738 nm and can be seen in Figure 1. The dye–surfactant complex formation is due to of the opposite charges of contributing moieties [21,22,23]. These complexes are different from their parent moieties and display an altered absorption spectrum. The micro-environment of dye molecules also affects their absorption spectrum [24,25]. Scheme 1 displays the interaction of NGB with CTAB and CPC.

**Figure 2:**
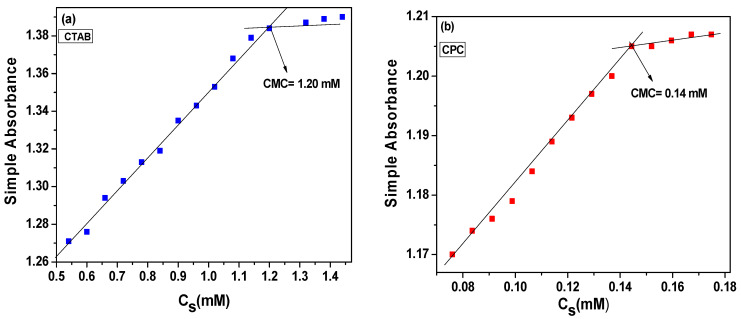
(**a**) Simple absorption of NGB as a function of concentration of CTAB, (**b**) simple absorption of NGB as a function of concentration of CPC.

The increased absorbance of NGB indicates the larger incorporation of dye molecules into the cationic micelle of CTAB and CPC. This absorbance increase is rapid up to CMC but after that almost becomes constant due to the maximum incorporation of dye molecules into a micelle. However, the gradual increase in absorbance even after the CMC is because of the dye molecules’ incorporation into recently formed micelles [26]. The plot of simple absorbance of NGB as a function of surfactant concentrations for CTAB and CPC are shown in Figure 2a,b, respectively, whereas such interactions were also studied using differential spectroscopy, as a very useful analytical technique for studying the dye–surfactant interactions. The increase in differential absorbance values predicts the strong dye–surfactant association. The deciding factors, such as polarity and hydrophobicity of molecules, influence the partitioning capability of NGB as a function of surfactant concentration. The increment in differential absorbance of dye with CTAB is an indication of the presence of dye molecules in a micelle. The polarizability of π-electronic clouds of aromatic compounds is also a significant factor in describing the strength of dye–surfactant interactions [27].

#### 2.1.2. Water–Micelle Partitioning of NGB

The partition coefficient not only explains the solubilization mechanism but also comprehends several industrial applications, such as separation by solubilization followed by ultrafiltration. K_x_ is the ratio of concentration (in mole fraction) of the dye molecules in micelle to that in the bulk aqueous phase. The partition of dye molecules between the micellar and aqueous phases is governed by partition law. The value of the partition coefficient is computed from data of differential absorbance as reported by Kawamura et al. Gibbs partitioning energy has negative values which confirm the spontaneity of the solubilization process.

Surfactant concentration and dye–surfactant ion pairs are directly proportional. Micellization is a starting point for dye encapsulation which is evident from a break in the absorption curve and is displayed in Figure 2a,b. These figures are indicative of the relation of dye loading, surfactant concentration, and CMC values of CTAB and CPC. The CMC values for CTAB and CPC are 1.20 and 0.14 mM, respectively. Cationic anionic complexes suppress the repulsion between the surfactant heads which also lead to CMC value reduction for both the surfactants. With a further increase in surfactant concentration after surfactant-induced redshift, there is no observable change in λ_max_. This is evidence of there being no change in dye–surfactant interactions in the pre- and post-micellar region [25]. The presence of the surfactant at a critical micellar concentration (CMC) in dye solution led to a hyperchromic shift in dye spectra and is evident in the monomeric encapsulation of dye [28,29].

**Figure 3:**
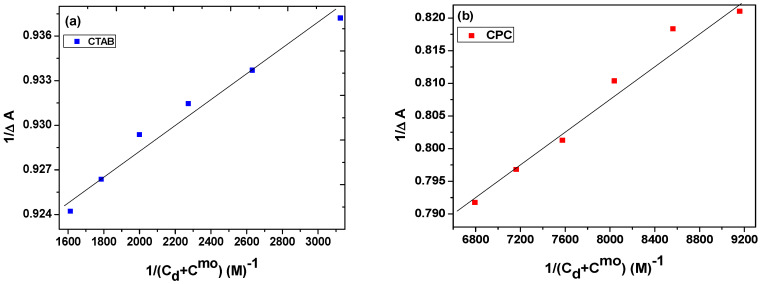
(**a**) Calculation of partition constant, K_x_ by Kawamura equation, for NGB-CTAB, (**b**) calculation of partition constant, K_x_ by Kawamura equation, for NGB-CPC.

Micelle hosts dye molecules in the outer palisade layer where water molecules exist in abundance. Dyes and other chromophore groups interact with surfactant head groups via coulombic forces and are also able to effectively absorb light. Differential spectroscopy can be employed for the quantitative study of the degree of dye–surfactant interaction and micelle–water partitioning. The data of the differential spectroscopy in the presence of CTAB and CPC are plotted in Figure 3a,b. The value of partition constant k_c_ is calculated from Figure 3a,b for the employed systems. Using the partitioning constant, a change in Gibbs energy is calculated. The calculated parameters are summarized in Table 1 and Table 2 in the presence of CTAB and CPC, respectively. The spontaneous interaction of dye–surfactant and water–micelle partitioning is evident from negative values of change in Gibbs free energy. The observed results are evidence of greater dye dispersion spontaneity in the heterophase system than the dye–surfactant interaction.

#### 2.1.3. Effects of TX-100 on the Partitioning of NGB

For the solubilization process, mixed micellization is more efficient as compared to a single surfactant system. The literature reported single micellar systems as ineffective for homogeneous dispersion of hydrophobic moieties [24]. Mixed micellization has been performed by both of the cationic surfactants individually by taking five different concentrations of, i.e., 0.09 mM, 0.11 mM, 0.13 mM, 0.15 mM, and 0.17 mM, to assess the potential of non-ionic surfactants for dye intercalation enhancement in cationic/nonionic surfactant systems. TX-100 molecules arrange themselves between the charged (cationic) head groups of CTAB and CPC and hence decrease the repulsion between these ionic heads. Due to the formation of the large size of the micelle, more dye is solubilized inside the micelle, and so enhanced solubilization occurs. Sometimes, after a specific rise in the concentration of nonionic surfactant, the value of partition coefficient and Gibbs free energy decreases due to the decrease in the ionic character in the mixed micelle and is also responsible for the weakening of the electrostatic interaction between the NGB and mixed micelle. The negative sign in ΔG_p_ represents the spontaneous behavior of NGB partitioning in a mixed micellar system [30]. Several spectral parameters of NGB dye have been detected by conducting several absorption experiments under varying Triton X-100 concentrations in terms of mixed micelles. These parameters are reported in Table 1 and Table 2 for CTAB and CPC, respectively.

In a mixed micellar system of a cationic surfactant and TX-100, the concentration of TX-100 and partition coefficient have a fine relationship. A minute addition of TX-100 in CTAB or CPC at micellar concentration was found to enhance the solubility more than the single micellar system of cationic surfactants. The synergism of cationic and non-ionic surfactants is behind this enhanced solubilization power of this mixed micellar system. The addition of two surfactants in the same solvent leads to stable mixed micelle formation instead of individual surfactant micelle formation [31,32]. Cationic and non-ionic surfactants arrange themselves alternately in mixed micelles to nullify the repulsion between similarly charged head groups of cationic surfactants and lead to the formation of mixed micelles of greater stability than the individual surfactant micelles [33].

**Scheme 2:**
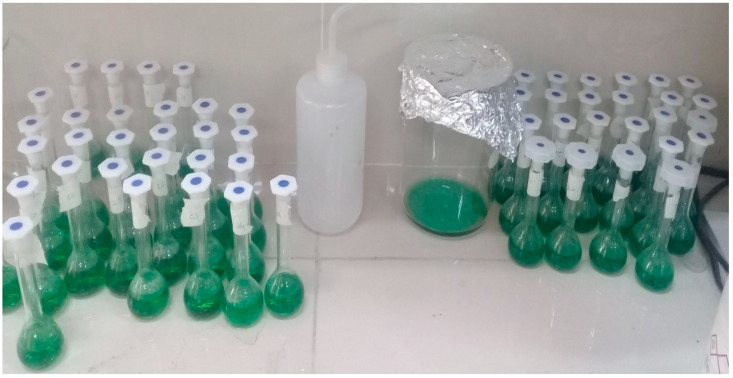
Different formulations of NGB in the presence of CTAB and CPC.

In addition to having enhanced stability, mixed micelles bear the properties of individual participants. Enhanced feasibility of dye/hydrophobic solute shifting from bulk aqueous media to micelle is associated with mixed micellization. The mixed micellar approach enhances dye–surfactant interaction for longer entanglement of dyes in micelle as well as the carrying capacity of the micelles. The value of the partition coefficient increases with an increase in the concentration of TX-100 until the setting of a suitable concentration. Scheme 2 represents the different formulations of NGB in the presence of CTAB and CPC.

On mixing, a subsequent decrease in solubilization is because of suppressed surface charge density. Low ionic potential suppresses the dye–surfactant binding and storing capacity of a mixed micelle. The quantitative expression of the storage capacity of a micelle is K_x_, and the suppression of charge density will release a substantial amount of dye from the micelle [34,35].

The increase in packing parameters led to sphere-to-rod-like shape transition in micelles. This enhanced packing parameter is because of suppressed cationic surfactant head groups in mixed micelles caused by nonionic surfactant molecules [36,37]. The negative charge of Naphthol Green B reduces the repulsion between CTAB/CPC’s cationic head groups and facilitates micellization. The potential of negatively charged sulfate groups of hydrophobic moieties to morphologically alter micelles is also reported in the literature [37]. The calculations given in Table 1 and Table 2 explain the influence of a nonionic surfactant on the solubilization power, and the value of Gibbs energy increases from −34.05 kJ mol^−1^ for CTAB to −39.65 kJ mol^−1^ at 0.13 mM concentration of TX-100 for CTAB/TX-100 and −37.62 kJ mol^−1^ for CPC to −47.94 kJ mol^−1^ at 0.11 mM concentration of TX-100 for CPC/TX-100, hence, providing a quantitative depiction.

### 2.2. Mechanism of Micellar-Enhanced Ultrafiltration of NGB by CTAB and CPC

In the MEUF process, a micellar solution is used at a concentration greater than the critical micellar concentration (CMC) to solubilize the dye, so, due to the opposite nature of pollutant and surfactants, the pollutant molecules are entrapped by the micelles and, therefore, the true solution is transformed into the colloidal solution. Due to the encapsulation of the pollutant (NGB) by the micelle, the size of the pollutant has been increased and micelles containing pollutants can be successively removed by an ultrafiltration membrane [7]. Although the pores of the membrane may become blocked by the pollutant molecules, it has been observed that there is no need to change the membrane after each run and it has even been found to be stable and capable of working for multi-pollutant system as well. In this study, a single membrane has been used which is then regenerated by washing via applying pressure ranging between 3–5 bars, so that the membrane is ready to be reused for the filtration process.

#### 2.2.1. Calculation of Rejection Percentage (R%) and Permeate Flux (J)

##### Effect of Concentration of Surfactants

**Figure 4:**
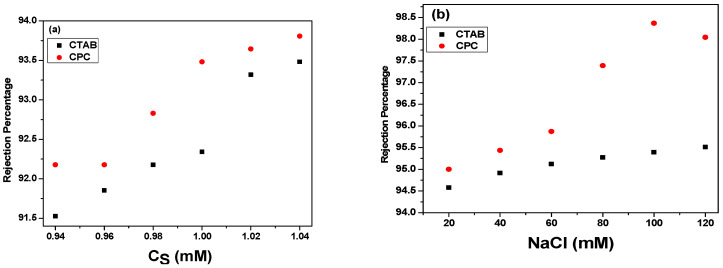

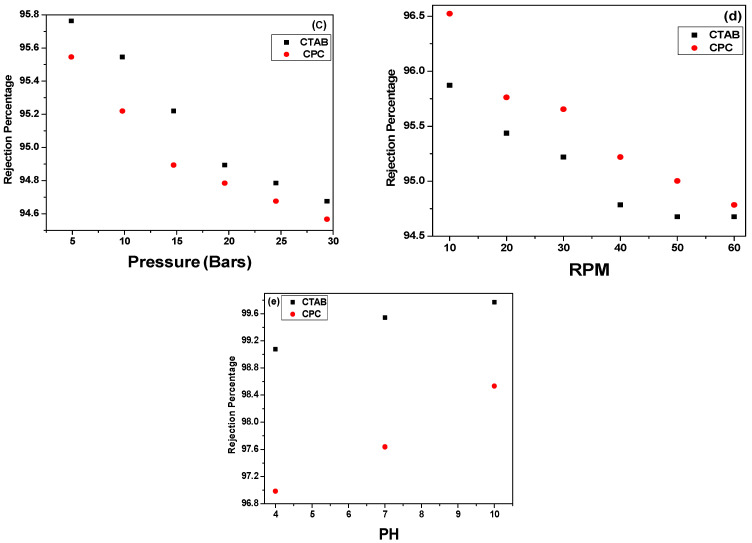
Rejection percentage (R%) for the effect of (**a**) surfactant concentration (C_S_), (**b**) electrolyte concentration (NaCl), (**c**) transmembrane pressure, (**d**) RPM using (**e**) pH.

To study the removal efficiency of NGB, two cationic surfactants, CTAB and CPC, were used. At fixed values of room temperature (25 °C), 20 bar pressure, 10 RPM stirring speed, and 0.03 mM concentration of NGB, the different concentrations of CTAB and CPC, ranging from 0 to 1.04 mM and 0 to 0.25 mM, respectively, were observed. The dye removal ability of CPC and CTAB corresponds with the Stern layer of their cationic micelles attracting the oppositely charged anionic dye with a sulfate group. Electrostatic interactions are operative between micelles and NGB molecules. The concentration of surfactants, number of available sites to trap anionic dye and ultimately the rejection percentage are directly related. A large amount of dye molecules were encapsulated and then rejected by the ultrafiltration membrane [7,38]. The results in Figure 4a and Table 3 demonstrated the increase in R% by increasing the concentrations of both surfactants, but greater rejection of NGB is observed by CPC as compared to CTAB. The rejection percentage calculated for CTAB and CPC was found to be 93.48% and 93.80%, respectively.

**Figure 5:**
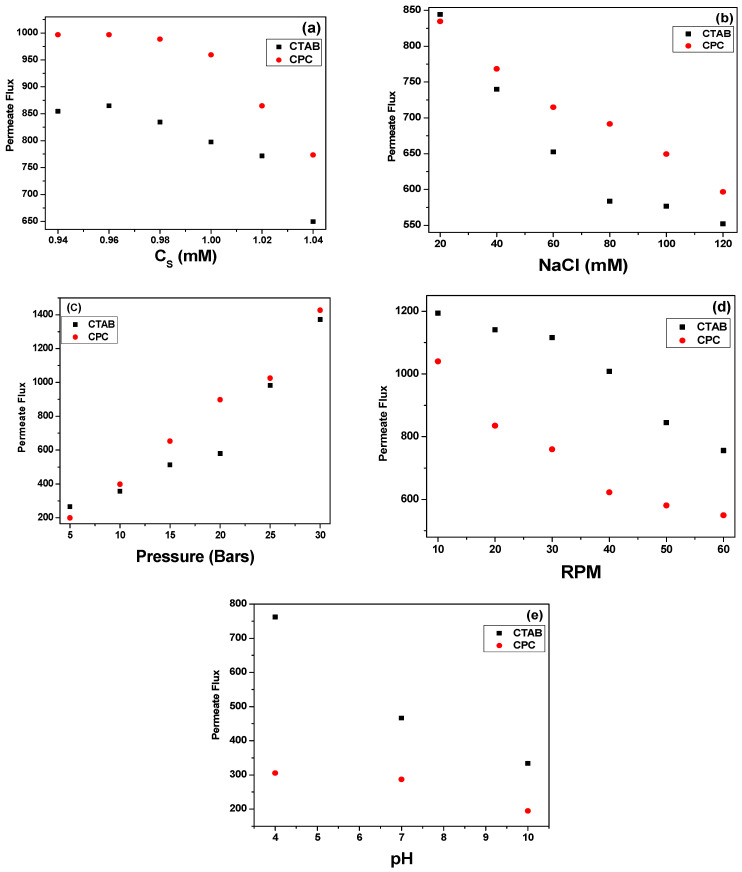
Permeate flux (J) for the effect of (**a**) surfactant concentration (C_S_), (**b**) electrolyte concentration (NaCl), (**c**) transmembrane pressure, and (**d**) RPM, (**e**) pH.

For the same experiment above, the concentration of CTAB and CPC is observed to have an inverse relation with the permeate flux. The minimum flux values obtained were 1669 and 1025 J (L/hm^2^) at maximum surfactant concentrations (C_S_) of 1.04 mM and 0.25 mM for CTAB and CPC, respectively. This can be explained on the basis of the direct relation between the surfactant concentration, micelles and micellar retention by the membrane interface. Micellar accumulation on the membrane surface and the inside confined pore volume led to a decrease in flux [39]. The overall results for the changing concentrations of both CTAB and CPC are summarized in Table 3 and Figure 5a.

##### Effect of Electrolyte Concentration

Experiments have been performed with a distinct concentration of NaCl (electrolyte) to assess its impact due to the presence of a promising amount of industrial effluent. Due to the presence of a variety of pollutants, their removal proceeds through the formation of micellar complexes in MEUF. Salt, as an electrolyte, promotes counter ion production which decreases CMC by interacting with the micellar surface. This causes more hindrance for dye molecules by disrupting their passage [7]. This is the reason that an increase in the rejection percentage was observed by varying its concentration from 20–120 mM for both CTAB and CPC while keeping all other parameters constant. High salt concentrations decrease the repulsion between charged micelle heads, leading to an increase in the number of counter ions, and thus an enhanced aggregation number of micelles. The maximum rejection percentages for CTAB and CPC were observed at 120 mM and 100 mM and found to be 95.51% and 98.37%, respectively, as shown in Figure 4b and Table 4. The electrolyte favors micellization and expands the concentration polarization effect, consequently resulting in flux decline. NaCl promotes aggregation, leading to a decrease in the dye solubility in the solvent [40].

Increasing values of NaCl from 20 to 120 (mM) decreased permeate flux due to the accumulation of micelles at the surface of the membrane causing hindrance to the flow across the membrane. Results are plotted in Figure 5b and summarized in Table 4 for CTAB and CPC, respectively.

##### Transmembrane Pressure

Transmembrane pressure (TMP) is basically an external applied pressure or the existence of pressure gradient across the feed and the permeate side [7]. The TMP pressure for both CTAB and CPC was varied from 5, 10, 15, 20, 25, and 30 bars while keeping the other parameters constant. A smooth decrease in the rejection coefficient is illustrated in Figure 4c. Pressure increase overcomes membrane resistance leading to an enhanced solution circulation through the membrane [7]. The acquisition of NGB molecules by micelles decreases with an increase in pressure. The significant reason for this is that the solubilization of NGB molecules by surfactant micelles is also lower with increasing pressure.

The rejection of NGB was observed to be lesser at high pressure [41]. Therefore, CTAB and CPC show their best rejection values of 95.76% and 95.54 % at a pressure value of 5 bars, as shown in Table 5. While at 30 bars, the solubilization of micelle capacity and stability on the surface of the membrane decrease due to micelle distortion. Thus, comparatively low rejection has been observed. Following Darcy’s Law, the pressure was varied from 5 to 30 bars to observe the flux. According to this law, flux is the product of membrane permeability (Lp) and change in pressure. An increase in flux value from 5 to 30 bars led to an increase in the speed of solution processing by the membrane. This increment confirms the process to be in a pressure-controlled region having no effect on concentration polarization. Figure 5c displays the permeate flux for CTAB and CPC with values 265–1372 and 199–1426 J(L/hm^2^) at 5 to 30 bars, respectively. The tabular form is given in Table 5.

##### RPM

The revolutions per minute (RPM) is basically the number of turns per minute or number of times something moves round in a minute, and here the values have been varied from 10 to 60 RPM. Rejection values were observed to be decreased from lower to higher rotation speed as presented in Figure 4d for CTAB and CPC. At 10 RPM, the exceptional values of rejection were calculated as 95.87% and 96.52% for CTAB and CPC, respectively. Micelles containing NGB molecules are larger and can easily be filtered by an ultrafiltration membrane of 10,000 MWCO, however, rapid stirring initiates de-micellization. High RPM decreases micelle size, so the micelles incorporating dye molecules are interrupted, eventually allowing them to pass through the membrane. Therefore, a decrease in the rejection percentage was observed at low pH, as shown in Table 6.

RPM and permeate flux are inversely related, as evident from the data presented in Table 6 and plotted in Figure 5d, according to which permeate flux decreases from 10 to 60 RPM for both CTAB and CPC. The decline is attributed to de-micellization as high RPM compels the individual monomers to penetrate the membrane pores, blocking the flow path and decreasing the efficiency of the process [7].

##### Effect of pH

The effect of pH was assessed at pH values 4, 7 and 10 by keeping other parameters constant for both CTAB and CPC solutions. The maximum rejection was observed to be at high pH values, i.e., 99.77% and 98.53% for CTAB and CPC, respectively, and has a close agreement with the literature [42]. Contrary to that, permeate flux was observed to be decreased at higher pH. The observed decline in the rejection can be explained on the basis of competition between the cations and H^+^. Protonation is behind the decrease in micellar and cation interactions at low pH. Therefore, pH has been earmarked for further work. The results are given in Table 7 and plotted in Figure 4e [42,43]. Due to the concentration polarization effect, the permeate flux decreases from pH value 4 to 10 as shown in Figure 5e.

##### Comparison among Effects of Various Factors

Both surfactants were found to be good candidates for solubilization and subsequently the MEUF process, but it has been noticed that in the majority of the cases, owing to the greater rejection, CPC is more efficient than CTAB.

## 3. Parameter Calculated

### 3.1. Partition Constant and Gibbs Energy of Partition

There are micellar and bulk aqueous mediums in which the dye molecules are separated. Based on the partition coefficient, the Kawamura equation can be used to quantify relative dye molecule distributions between the two phases (1) [44,45].

(1) $$\frac{1}{\Delta A}=\frac{1}{K_{c}\;{\Delta A}_{\infty }\left(C_{d}+C_{smo}\right)}+\frac{1}{{\Delta A}_{\infty }}$$

In the above equation, *C_d_* denotes the molar concentration of NGB in mol dm^−3^, and *C_s_^mo^* represents the analytical concentration of surfactants (2)

(2) $$C_{s}^{mo}=C_{s}-CMC_{o}$$

where *CMC_o_* is the critical micelle concentration of surfactants in the absence of dye and *C_s_* is the total molar concentration of surfactant. The differential absorbance under experimental circumstances is represented by the symbol “ΔA”, which remains unchanged during infinite dilution.

The partition coefficient *k_x_* has no units, while *K_c_* is the partition constant with units of dm^3^ mol^−1^. *k_x_* is the product of *K_c_* and the number of moles of water per liter (nw), where nw is the multiple of *K_c_*. Gibbs energy of the partition is calculated using Equation (3) [30].

(3) $${\Delta G}_{p}=-RTlnk_{x}$$

### 3.2. Permeate Flux

To check the efficiency of MEUF, changes in permeate flux (*J*) values are determined. The micelles layer starts depositing on the membrane interface causing hindrance in cross flow, eventually decreasing the permeate flux. The following equation can be applied to measure the amount of flux.

(4) $$J=\frac{V}{t\times A}$$

Here, “*A*” is the effective area of the membrane, “*V*” is the volume of permeate solution, and “*t*” is the time taken for ultrafiltration [46].

### 3.3. Rejection Percentage

In addition to the above-mentioned criteria, an important way to monitor the efficiency of both processes is by calculating a parameter referred to as the rejection percentage (R%). In MEUF, as micelles form above the CMC of surfactant, dye molecules solubilize within these micelles causing their size to increase and then are ultimately rejected by ultrafiltration membranes. The rejection percentage is calculated to demonstrate how much dye (NGB) was removed. The equation is written as

(5) $$R\;\left(\%\right)=\left[1-\frac{C_{p}}{C_{F}}\right]\times 100$$

In this equation, *C_p_* and *C_F_* are the concentrations of pollutants in the permeate and feed solution, respectively [7].

## 4. Materials and Methods

### 4.1. Materials Used

**Scheme 3:**
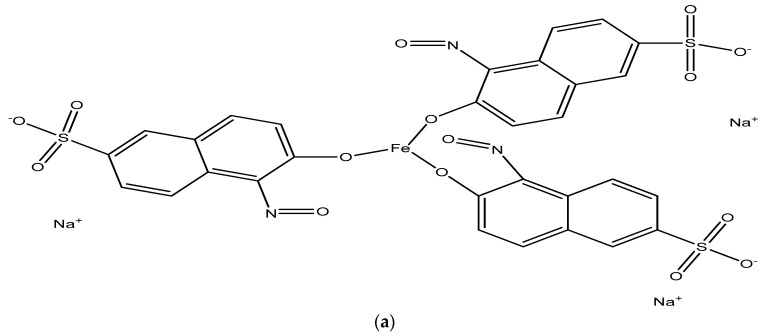

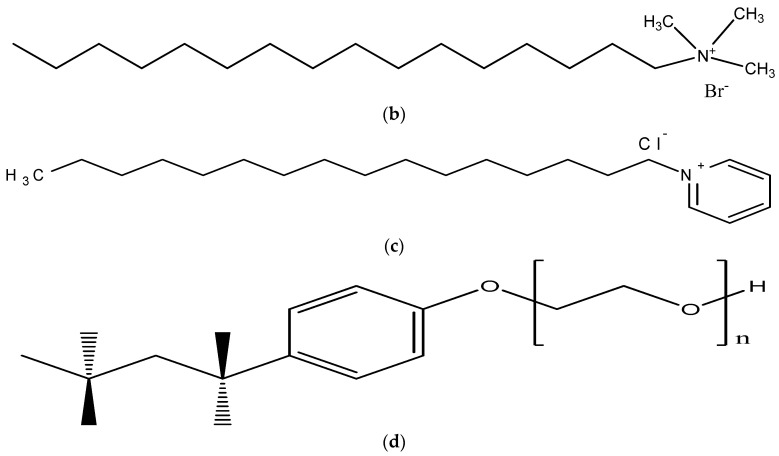
The structures for (**a**) NGB (**b**) CTAB (**c**) CPC (**d**) TX-100.

Anionic dye, Naphthol Green B (NGB, C_30_H_15_FeN_3_Na_3_O_15_S_3_) was purchased from Sigma–Aldrich. The cationic surfactants; cetyltrimethylammonium bromide (CTAB, C_16_H_33_N(CH_3_)_3_Br) and cetylpyridinium chloride (CPC, C_21_H_38_CNl,) were also purchased from Sigma–Aldrich and used as received. Both surfactants have a percentage purity of 99.9%. A nonionic surfactant Triton X-100 (TX-100) with a percentage purity of 98% was purchased from the Daejung company and used for the mixed micellar formulations. The chemical structures of these chemicals are presented in Scheme 3 and some necessary details are summarized in Table 8. All the solutions were prepared in distilled water. An ultrafiltration membrane of 10,000 MWCO of regenerated cellulose was acquired from Amicon Bioseparations (EMD) Millipore corporation, Burlington, MA, USA.

### 4.2. Experimental Procedure

A double-beam UV/visible spectrophotometer equipped with a Xenon lamp purchased from Peaks C-8200S, made in the USA, was used to record the absorption spectra [47,48]. Initially, a stock solution of NGB of 1 × 10^−4^ M concentration was prepared in distilled water. A series of different concentrations of CPC and CTAB were also prepared in a ternary mixture environment, with surfactant concentrations ranging from sub-micellar to micellar concentrations using a nonionic surfactant (TX-100). The mixed micellar solutions were prepared as CTAB/NGB and CPC/NGB in water. The CTAB/TX-100 and CPC/TX-100 systems were studied by making different formulations of mixed micellar media. A simple and differential absorption spectrum was recorded spectroscopically.

Membrane stirred cell (Amicon 8400 Millipore, (625 Bunker Ct, Vernon Hills, IL, USA) with cell capacity 350 mL, effective membrane area 0.418 cm^2^, and maximum operating pressure 50 bars was used for ultrafiltration. The membrane was soaked in distilled water for 24 h before process startup and then washed with deionized water after each run. At room temperature, the RPM and TMP values varied from 10–60 RPM and 5–30 bars, respectively. Optical analysis of feed and permeate solutions was performed using a UV/visible spectrophotometer. The feed solution is basically the solution containing pollutant, surfactant or any additives which are to be added to the filtration cell, whereas the permeate solution contains the pollutant-free solution but may not be 100% (may contain some of the pollutant molecules and some free surfactant monomers). Due to the encapsulation of pollutant (NGB) by the micelle, the size of the pollutant has been increased and micelles containing pollutants can be successively removed by an ultrafiltration membrane. The effect of various parameters including dye, surfactant and electrolyte concentrations, pressure, pH, and RPM were determined [7].

## 5. Conclusions and Future Perspectives

Industry demands an explanation of both the theoretical and practical facets of solubilization. This study aimed to provide an optimum surfactant formulation for Naphthol Green B solubilization and micellar-enhanced ultrafiltration. The change in the UV/visible absorption spectra of NGB with a change in concentration of surfactant is dependent on the nature of the surfactant. With an enhanced hydrophobicity of the mixed micellar system, an increase in dye molecules and micelles is observed. The addition of nonionic surfactant to the solution of cationic surfactant was found to alter the solubilization as well as the physicochemical properties. The influence of Coulombic forces along with the London dispersion forces is evident from the difference in solubilization power, despite the similar hydrophobic chain length of CTAB and CPC. Spontaneous dye partitioning was proven by the experimental results. The micellization of CTAB and CPC is an enthalpy and entropy driven spontaneous processes.

In conclusion, mixed micellar systems are found to have greater efficiency than individual micellar systems for the stated surfactants. Micellar-enhanced ultrafiltration can be employed in multiple wastewater treatments, including dye removal, as a versatile technique. MEUF was assessed for the removal of Naphthol Green B from the aqueous system. Multiple operational parameters were examined to optimize the removal of NGB by the dual-micellar system of CPC and CTAB.

The effects of surfactant concentration, transmembrane pressure, agitation speed (RPM), electrolyte concentration (NaCl), and pH were investigated. Rejection rate and permeate flux were used to check the efficacy of this process. The removal efficiency was high for both surfactants. High surfactant concentration, low pressure, low RPM, high pH, and high electrolyte concentration all gave the highest levels of rejection. The maximum removal by CTAB and CPC was 99.77 % and 98.53%, while the highest flux was 1669 J(L/hm^2^) and 1025 J(L/hm^2^), respectively.

In future, MEUF will be used at a pilot scale. This study opens the door to further effective research in future. The significant results obtained revealed it to be a successful methodology if employed by industries to keep this environment clean. Other kinds of pollutants would also be considered in future studies along the combination of dyes and metals. Selective studies on plants and soil tests will also establish the extent of the toxicity in our crops and land. Methodology will be developed to treat a pollutant dense mass in a controlled environment.

## Figures and Tables

**Table 1:** Gibbs energy of partition ΔG_p_, partition coefficient, K_x_ partition constant, K_c_, for NGB/CTAB systems at various Triton X-100 concentrations.

[TX-100] (mM)	K_c_ × 10^−4^ (dm^3^ mol^−1^)	K_x_ × 10^−5^	ΔG_p_ (kJ mol^−1^)
0	1.67	9.30	−34.05
0.09	7.15	39.7	−37.64
0.11	1.01	56.3	−38.51
0.13	16.1	89.3	−39.65
0.15	4.36	24.2	−36.42
0.17	3.22	17.9	−35.67

**Table 2:** Gibbs energy of partition ΔG_p_, partition coefficient, K_x_ partition constant, K_c_, for NGB/CPC systems at various Triton X-100 concentrations.

[TX-100] (mM)	K_c_ × 10^−4^ (dm^3^ mol^−1^)	K_x_ × 10^−5^	ΔG_p_ (kJ mol^−1^)
0	7.1	39.4	−37.62
0.09	401	2230	−47.62
0.11	45.7	25.4	−47.94
0.13	57	317	−42.78
0.15	17.7	98.1	−39.88
0.17	1.12	6.24	−33.06

**Table 3:** Rejection coefficient, R%, and permeate flux, J, of Naphthol Green B for different surfactant concentrations, C_s_, at fixed dye concentration, C_F_, dye concentration in permeate solution, C_p_, filtration time, t, permeate volume, V_p_.

Sr. No.	C_S_ (mM)		C_F_ (mM)	C_p_ (μM)		R %		t (h)		V_p_ (ml)	J (L/hm^2^)	
	CTAB	CPC		CTAB	CPC	CTAB	CPC	CTAB	CPC		CTAB	CPC
1	0	0	0.02	9.26	1.63	53.71	29.92	0.184	0.154	50	1669	1025
2	0.94	0.15	0.02	1.69	1.56	91.52	92.17	0.155	0.138	50	712	996
3	0.96	0.17	0.02	1.63	1.56	91.85	92.17	0.150	0.124	50	684	996
4	0.98	0.19	0.02	1.56	1.43	92.17	92.82	0.143	0.121	50	649	988
5	1.0	0.21	0.02	1.53	1.30	92.34	93.48	0.138	0.119	50	626	959
6	1.02	0.24	0.02	1.34	1.27	93.31	93.64	0.140	0.120	50	595	864
7	1.04	0.25	0.02	1.30	1.24	93.48	93.80	0.136	0.116	50	584	773

**Table 4:** Rejection coefficient, R%, and permeate flux, J, of Naphthol Green B for different electrolyte concentrations, NaCl, at fixed dye concentration, C_F_, dye concentration in permeate solution, C_p_, filtration time, t, permeate volume, V_p_.

Sr. No.	[NaCl] (mM)	C_F_ (mM)	C_p_ (μM)		R %		t (h)		V_p_ (ml)		J (L/hm^2^)	
			CTAB	CPC	CTAB	CPC	CTAB	CPC	CTAB	CPC	CTAB	CPC
1	20	0.03	16.3	1.50	94.57	95.00	0.141	0.14	50	50	844	834
2	40	0.03	15.3	1.37	94.91	95.43	0.161	0.15	50	50	739	768
3	60	0.03	14.6	1.24	95.12	95.87	0.183	0.16	50	50	652	714
4	80	0.03	14.2	0.78	95.27	97.39	0.205	0.17	50	50	583	691
5	100	0.03	13.8	0.48	95.39	98.37	0.207	0.18	50	50	576	649
6	120	0.03	13.5	0.58	95.51	98.04	0.216	0.20	50	50	552	596

**Table 5:** Rejection coefficient, R%, and permeate flux, J, of Naphthol Green B for different values of pressure, at fixed dye concentration, C_F_, dye concentration in permeate solution, C_p_, filtration time, t, permeate volume, V_p_.

Sr. No.	Pressure (Bar)	C_F_ (mM)	C_p_ (μM)		R %		t (h)		V_p_ (ml)		J (L/hm^2^)	
			CTAB	CPC	CTAB	CPC	CTAB	CPC	CTAB	CPC	CTAB	CPC
1	5	0.03	1.27	1.34	95.76	95.54	0.45	0.60	50	50	265	199
2	10	0.03	1.34	1.43	95.54	95.21	0.33	0.30	50	50	356	398
3	15	0.03	1.43	1.53	95.21	94.89	0.23	0.18	50	50	512	652
4	20	0.03	1.53	1.56	94.89	94.78	0.20	0.13	50	50	579	897
5	25	0.03	1.56	1.60	94.78	94.67	0.12	0.11	50	50	981	1025
6	30	0.03	1.60	1.63	94.67	94.56	0.08	0.08	50	50	1372	1426

**Table 6:** Rejection coefficient, R%, and permeate flux, J, of Naphthol Green B for different values of RPM at fixed dye concentration, C_F_, dye concentration in permeate solution, C_p_, filtration time, t, permeate volume, V_p_.

Sr. No.	RPM	C_F_ (mM)	C_p_ (μM)		R %		t (h)		V_p_ (ml)		J (L/hm^2^)	
			CTAB	CPC	CTAB	CPC	CTAB	CPC	CTAB	CPC	CTAB	CPC
1	10	0.03	1.24	1.04	95.87	96.52	0.158	0.115	50	50	1194	1040
2	20	0.03	1.37	1.27	95.43	95.76	0.141	0.143	50	50	1141	834
3	30	0.03	1.43	1.30	95.21	95.65	0.118	0.157	50	50	1116	759
4	40	0.03	1.56	1.43	94.78	95.21	0.107	0.192	50	50	1008	622
5	50	0.03	1.60	1.50	94.67	95.00	0.104	0.205	50	50	844	581
6	60	0.03	1.60	1.56	94.67	94.78	0.100	0.217	50	50	755	549

**Table 7:** Rejection coefficient, R%, and permeate flux, J, of Naphthol Green B for different values of pH, at fixed dye concentration, C_F_, dye concentration in permeate solution, C_p_, filtration time, t, permeate volume, V_p_.

Sr. No	pH	C_F_ (mM)	C_p_ (μM)		R %		t (h)		V_p_ (ml)		J (L/hm^2^)	
			CTAB	CPC	CTAB	CPC	CTAB	CPC	CTAB	CPC	CTAB	CPC
1	4	0.03	2.27	1.21	99.07	96.98	0.157	0.235	50	50	761	305
2	7	0.03	1.37	0.94	99.54	97.63	0.256	0.250	50	50	466	287
3	10	0.03	0.68	0.58	99.77	98.53	0.358	0.368	50	50	333	194

**Table 8:** Required details of chemicals and materials used.

Chemicals/Materials	Nature	Molecular Weight g/mol	Source
Naphthol Green B	Anionic	878.4	Sigma–Aldrich
Cetyl trimethyl ammonium bromide (CTAB)	Cationic	364.44	Daejung, Korea
Cetyl pyridinium chloride (CPC)	Cationic	339.99	Daejung, Korea
Membranes	Regenerated cellulose of 10,000 MWCO	-	Amicon Bioseparations EMD Millipore Corporation
Ultrafiltration stirred cell	Pressure-controlled	-	Amicon 8400 Millipore, USA

## Data Availability

The data presented in this study are available on request from the corresponding author.

## Sample Availability

Samples of the compounds are not available from the authors.
